# Single‐cell RNA‐sequencing analysis reveals enhanced non‐canonical neurotrophic factor signaling in the subacute phase of traumatic brain injury

**DOI:** 10.1111/cns.14278

**Published:** 2023-06-02

**Authors:** Xuecheng Qiu, Yaling Guo, Ming‐Feng Liu, Bingge Zhang, Jingzhen Li, Jian‐Feng Wei, Meng Li

**Affiliations:** ^1^ Jiangsu Key Laboratory of Brain Disease Bioinformation, Research Center for Biochemistry and Molecular Biology Xuzhou Medical University Xuzhou Jiangsu China; ^2^ Department of Neurosurgery Xuzhou Hospital of Traditional Chinese Medicine Xuzhou Jiangsu China; ^3^ Department of Histology and Embryology Xuzhou Medical University Xuzhou Jiangsu China

**Keywords:** midkine, neural regeneration, neuroinflammation, pleiotrophin, prosaposin, traumatic brain injury

## Abstract

**Background:**

Traumatic brain injury (TBI) is a leading cause of long‐term disability in young adults and induces complex neuropathological processes. Cellular autonomous and intercellular changes during the subacute phase contribute substantially to the neuropathology of TBI. However, the underlying mechanisms remain elusive. In this study, we explored the dysregulated cellular signaling during the subacute phase of TBI.

**Methods:**

Single‐cell RNA‐sequencing data (GSE160763) of TBI were analyzed to explore the cell–cell communication in the subacute phase of TBI. Upregulated neurotrophic factor signaling was validated in a mouse model of TBI. Primary cell cultures and cell lines were used as in vitro models to examine the potential mechanisms affecting signaling.

**Results:**

Single‐cell RNA‐sequencing analysis revealed that microglia and astrocytes were the most affected cells during the subacute phase of TBI. Cell–cell communication analysis demonstrated that signaling mediated by the non‐canonical neurotrophic factors midkine (MDK), pleiotrophin (PTN), and prosaposin (PSAP) in the microglia/astrocytes was upregulated in the subacute phase of TBI. Time‐course profiling showed that MDK, PTN, and PSAP expression was primarily upregulated in the subacute phase of TBI, and astrocytes were the major source of MDK and PTN after TBI. In vitro studies revealed that the expression of MDK, PTN, and PSAP in astrocytes was enhanced by activated microglia. Moreover, MDK and PTN promoted the proliferation of neural progenitors derived from human‐induced pluripotent stem cells (iPSCs) and neurite growth in iPSC‐derived neurons, whereas PSAP exclusively stimulated neurite growth.

**Conclusion:**

The non‐canonical neurotrophic factors MDK, PTN, and PSAP were upregulated in the subacute phase of TBI and played a crucial role in neuroregeneration.

## Graphical abstract

Cell–cell communication analysis reveals that the canonical neurotrophic signaling (MDK, PTN, and PSAP) are upregulated rather than the canonical neurotrophic signaling in the subacute phase of TBI. Activated microglia promotes astrocyte‐secreting MDK, PTN, and PSAP, which enhances neural stem cell proliferation and neurite growth, and may induce spontaneous neuroregeneration.

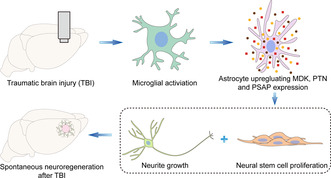

## 1 INTRODUCTION

Traumatic brain injury (TBI) usually results from external mechanical forces acting on the head. TBI disrupts the normal structure of the brain, leading to temporary or permanent brain impairment, and in severe cases, it may even cause death. Approximately 69 million individuals worldwide suffer from TBI annually.^1^ TBI has been recognized as a chronic disease; brain functions continue to deteriorate even years after the onset of TBI due to progressive neurodegeneration,^2^ leading to health deterioration and economic burden on the TBI survivors.^3^, ^4^ However, effective medical treatment for TBI is limited. Exploring the comprehensive molecular mechanisms of TBI is fundamental to developing novel therapeutic strategies for TBI.

Traumatic brain injury is classified into two categories according to the mechanism of injury: primary and secondary injury. Primary brain injury occurs immediately after a mechanical force is applied directly to the head, leading to acute neural cell death, hemorrhage, hematoma, and brain edema. Secondary injury is initiated after the primary insult and lasts for hours to even years.^5^ Several mechanisms contributing to secondary injury have been identified, including blood–brain barrier (BBB) disturbance, oxidative stress, free radical production, mitochondrial dysfunction, calcium‐mediated damage, inflammatory responses, excitatory amino acid neurotoxicity, and progressive cell loss.^5^, ^6^, ^7^ However, our understanding of the pathological mechanisms underlying TBI remains incomplete.

Previous studies have revealed changes in the genomic profile after TBI by utilizing heterogeneous mixtures of cell populations.^8^, ^9^ Expression of inflammation‐related genes is remarkably upregulated in the acute and subacute phases of TBI,^9^, ^10^ indicating that the immune response plays an important role in the early stages of TBI. Microglia, the resident immune cells of the central nervous system (CNS), upregulate genes associated with chemotaxis, such as C‐C motif chemokine ligand (CCL) and CXCL chemokines, as well as genes associated with cytokine signaling, such as interferon‐gamma and those belonging to the interleukin family.^10^ This event results in the recruitment of microglia from the peri‐injury region as well as the recruitment of peripheral immune cells to the injured sites in the brain during the acute phase of brain injury.^11^ Microglia can phagocytose cellular debris and are transformed to adopt anti‐inflammatory and pro‐regenerative phenotypes, leading to the secretion of anti‐inflammatory and neuroprotective factors to promote spontaneous recovery after TBI.^12^ Astrocyte represent another significant cell population involved in the pathogenesis of TBI. Astrocytes regulate the integrity of the BBB and the homeostasis of the CNS.^13^, ^14^ Astrocyte activation (reactive astrogliosis) has multiple roles in tissue remodeling processes after TBI, such as neurogenesis, synaptogenesis, BBB integrity, and glial scar formation.^15^, ^16^ Furthermore, the crosstalk between astrocytes and microglia also plays an important role in the development of CNS and diseases associated with it.^17^, ^18^ Bidirectional communication between microglia and astrocytes, mediated by the secretion of diverse signaling molecules, modulates their phenotype and biological functions reciprocally.^18^ However, the accurate mechanism of cell–cell communication in the subacute phase of TBI remains obscure.

Unbiased high‐throughput single‐cell RNA‐sequencing provides a novel perspective for exploring the molecular mechanisms of TBI. A recent study revealed changes in the cell type‐specific genes and pathways in the hippocampus during the acute phase (within 24 h) of concussive brain injury using single‐cell RNA‐sequencing.^19^ However, the detailed cell–cell communication and single‐cell molecular alterations in the subacute phase of TBI remain unclear.

Using high‐throughput single‐cell RNA‐sequencing technology, we sought to identify the cell populations involved and changes in the signaling pathways in the subacute phase of TBI at the single‐cell level. In this study, we found that cell–cell interactions were significantly increased in astrocytes, microglia, and oligodendrocytes. We found that the signaling of three non‐canonical neurotrophic factors, midkine (MDK), pleiotrophin (PTN), and prosaposin (PSAP), was significantly upregulated, especially in astrocytes. These findings were validated in a time‐course study using a rodent model of TBI. We then investigated the possible roles of MDK, PTN, and PSAP in the pathogenesis of TBI and found that they played a limited role in inflammatory regulation but significantly increased neurite growth and neural progenitor cell (NPC) proliferation. Our findings provide novel insights into the pathology of TBI, paving the way for the development of potential treatments for TBI.

## 2 METHODS

### 2.1 Single‐cell RNA‐sequencing data analysis

The single‐cell RNA‐sequencing data of TBI (fluid percussion injury model) were collected from Gene Expression Omnibus (GEO, accession number GSE160763).^20^ The datasets from the control and TBI group were integrated and analyzed using the Seurat R package (version 4.1.1).^21^ The number of genes detected in each cell that was smaller than 200 and the percentage of mitochondrial genes that was larger than 5% was filtered. The dataset was normalized, and the most highly variable genes were identified using default parameters of the “NormalizeData” and “FindVariableFeature” functions, respectively. We then performed PCA and selected the top 20 principal components (PCs) according to the “Elbow” plot. The cell clusters were then identified using the function of “FindClusters” (resolution = 0.2). To visualize the cells, T‐distributed Stochastic Neighbor Embedding (t‐SNE) was performed using the top 20 PCs. To find the marker genes of the different clusters, the function of “FindAllMarkers” (min.pct = 0.25, logfc.threshold = 0.25) was used and the top 10 highly expressed differential genes were used for cell type annotation (Table S1). The cell type annotation was based on the known cell type‐specific markers from previous studies and CellMarker (http://xteam.xbio.top/CellMarker/).^22^ The Cell–cell communication analysis was performed using the R package of CellChat (version 1.1.3) according to the tutorial.^23^

### 2.2 Traumatic brain injury model

Three‐month‐old male mice (C57BL/6) were anesthetized with Avertin (Sigma‐Aldrich, 250 mg/kg body weight, i.p.). The mice were fixed on a stereotaxic frame, and a 4‐mm‐diameter cranial window centered at 2 mm lateral to the Bregma was performed on the right side of the skull. TBI was induced using a weight drop model. A steel block weighing 45 g with a 3‐mm‐diameter flat tip was dropped onto the exposed cortex from a height of 30 cm with an impact depth of 2 mm. The mice in the sham group were handled in the same manner but without craniotomy and impact. After surgery, mice were placed on a homoeothermic blanket (37°C) until complete recovery from the anesthesia. An analgesic (meloxicam, 10 mg/kg) was subcutaneously injected for postoperative pain control.

### 2.3 Tissue processing and histological immunostaining

The mice were sacrificed at 2 days (TBI‐2d), 7 days (TBI‐7d), 14 days (TBI‐14d), and 30 days (TBI‐30d) after TBI. Mice were anesthetized with a lethal dose of Avertin, and then transcardially perfused with 0.01 M phosphate‐buffered saline (PBS) containing 10 U/mL of heparin, followed by 4% paraformaldehyde (PFA) solution. Brains were collected and post‐fixed in 4% paraformaldehyde solution overnight at 4°C and then soaked into 30% sucrose in PBS for dehydration. Brains were sectioned using a cryostat (Leica Biosystems) with 20‐μm thickness when mice brains sunk in the 30% sucrose solution. Brain sections were stored in cryoprotectant (0.01 M PBS with 30% ethylene glycol and 30% glycerol, pH 7.2) at −30°C until use.

Two to three brain sections from Bregma +1 mm to −0.5 mm were chosen for free‐floating immunofluorescence staining. Brain sections were rinsed with PBS and blocked in 10% normal goat/donkey serum in 0.3% Triton X‐100 PBS for 1 h at room temperature (RT) before incubation with primary antibody (Table S2) overnight at 4°C. For negative control sections, the primary antibodies were omitted. The following day, sections were rinsed three times with PBS and incubated with corresponding secondary antibodies (Table S2) at RT for 2 h. After three times rinsing, brain sections were counterstained with 4′,6‐diamidino‐2‐phenylindole (DAPI) and mounted on the slides.

To quantify MDK, PTN, and PSAP expression in the peri‐injured site of the cortex, the integrated density of the positive area was analyzed using Fiji software and then normalized by the sham group. The percentage of GAP43 positive was also analyzed using Fiji with a fixed threshold. To evaluate the number of GAP43 positive puncta, a Fiji plugin of “Find Maxima” with 15 of “prominence” was used after subtracting the background of images with 50 pixels of rolling ball radius.

To quantify the MDK, PTN, and PSAP expression in different cell types at 7 days after TBI, brain sections were co‐immunostained with NeuN (neuronal marker), IBA1 (microglial marker), or GFAP (astroglial marker). The percentage of the co‐stained area (per field) was analyzed using Fiji software. The co‐stained area was also normalized by the corresponding positive area of the neural cell markers.

### 2.4 Western blotting assays

The astrocyte was cultured from 1‐day postnatal mice.^24^ The primary cultured astrocyte and BV2 cell line were treated with lipopolysaccharide (LPS, 1 μg/mL) for 24 h. To evaluate the effect of the activated microglia on the expression of MDK, PTN, and PSAP in astrocytes, the conditional medium from LPS‐stimulated (1 μg/mL for 48 h) BV2 cells (LPS‐BV2‐CM) was collected and added into primary cultured astrocyte for 24 h. The conditional medium from BV2 cells without LPS stimulation (BV2‐CM) was used as a control. Cells were then lysed in ice‐cold Radio Immunoprecipitation Assay (RIPA) Lysis buffer with a proteinase inhibitor cocktail. The lysates were centrifuged at 12,000 rpm for 15 min at 4°C. The supernatant was collected and quantified using a BCA protein assay kit (Beyotime). For Western blot assay, the quantified samples were mixed with loading buffer and boiled for 10 min. The samples were electrophoresed in 12% SDS‐PAGE gel and transferred to a nitrocellulose membrane (0.45 μm pore size, Amersham Biosciences GE). The blotting membrane was rinsed with Tris‐buffered saline containing 0.5% Tween‐ 20 (TBST) and blocked with 5% non‐fat milk for 1 h at RT and probed overnight at 4°C with primary antibodies (Table S2). After rinsing with TBST, the blotting membrane was incubated with the corresponding horseradish peroxidase (HRP) conjugated goat anti‐mouse IgG or goat anti‐rabbit IgG (1:10,000, Proteintech) for 2 h (RT). The membranes were then washed with TBST, incubated with Chemiluminescent HRP substrate (Millipore), and visualized using a FluorChem FC3 Imaging System (Protein Simple).

### 2.5 Real‐time quantitative polymerase chain reaction

To detect the effect of MDK, PTN, and PSAP on inflammatory‐related genes expression, BV2 cells were treated with MDK (50 ng/mL, Sangon Biotech), PTN (50 ng/mL, Sangon Biotech), and TX14(A) (a prosaposin‐derived peptide, 500 nM, MCE), or co‐stimulated with LPS (500 ng/mL) for 24 h. Total RNA was isolated using TRIzol. The complementary DNA (cDNA) was synthesized via reverse transcription using PrimeScript™ RT reagent Kit (TaKaRa). The inflammatory‐related gene expression was evaluated using real‐time quantitative polymerase chain reaction (qPCR) using a 2× Universal SYBR Green Fast qPCR Mix kit (ABclonal) according to the instruction manual. The primers are shown in Table S3.

### 2.6 The proliferation of human iPSCs‐derived neural progenitor cells and neurite growth of human iPSCs‐derived neuron assay

To induce human‐derived induced pluripotent stem cells (iPSCs) to differentiate into neural progenitor cells (NPCs) and neurons, feeder‐free iPSCs were maintained in mTeSR™ Plus medium (STEMCELL) and then changed to neural induce medium (1:1 mixture of DMEM/F12 and Neurobasal, 1% N2 Supplement, 2 mM L‐ Alanyl Glutamine, 10 μM SB431542, 100 nM LDN193189) for 9 days (daily medium change). Then, cells were dissociated with TrypLE (Thermo Fisher Scientific) and plated on the low growth factor Matrigel Matrix‐coated dishes in NPCs medium (1:1 mixture of DMEM/F12 and Neurobasal, 1% N2 Supplement, 0.5% B27 Supplement without vitamin A, 2 mM L‐Alanyl Glutamine) with ROCK inhibitor Y27632 (10 μM, MCE) at the first day. The NPC medium was changed daily until neural rosettes formed to acquire NPCs. The NPCs were dissociated with Accutase (STEMCELL) and cryopreserved in liquid nitrogen until use.

To evaluate the role of MDK, PTN, and PSAP on the proliferation of human iPSCs‐derived NPCs, the NPCs were seeded on low growth factor Matrigel Matrix‐coated 24‐plate wells in NPCs medium with ROCK inhibitor Y27632. The next day, the medium was changed into fresh NPCs with/without MDK (50 ng/mL), PTN (50 ng/mL), and TX14(A) (500 nM) for 16 h, and then EdU (10 μM) was added for another 8 h. The cells were fixed in 4% PFA. The Edu‐labeled cells were detected using the BeyoClick™ EdU‐594 cell proliferation kit according to the manual instruction. The EdU‐positive cells were counted manually using Fiji software, and the percentage of EdU‐positive cells was analyzed.

iPSCs‐derived neurons were used to evaluate the effects of MDK, PTN, and PSAP on neurite growth. A 24‐well plate was coated with 0.1% polyethyleneimine (Sigma‐Aldrich) in 0.15 M borate buffer (1.86 g boric acid, 5.4 g sodium tetraborate decahydrate, in 500 mL distilled water, pH 8.5) for 1 h. After rinsing with distilled water, the plate was coated with low growth factor Matrigel Matrix (Corning) for 1 h. iPSCs‐derived NPCs were plated on the treated 24‐well plate in neuron culture medium (1:1 mixture of DMEM/F12 and Neurobasal, 1% N2 Supplement, 0.5% B27 Supplement, 2 mM L‐Alanyl Glutamine, 3 g/L glucose, 200 μM L‐ascorbic acid, and 1 μM cAMP) with ROCK inhibitor Y27632. The next day, the medium was changed to fresh neuron culture medium with/without MDK (50 ng/mL), PTN (50 ng/mL), and TX14(A) (500 nM) and kept in culture for another 72 h. The neurons were fixed with 4% PFA and immunostained with mouse anti‐Tuj1 (1:500, Beyotime) to visualize neurites. The total length of neurite per cell was analyzed using the Macro tool of “Measure_Skeleton_Length_tool” via Fiji software.^25^

### 2.7 Statistical analysis

Data were presented as mean ± SEM or median with interquartile range (in box plots). The assumption of equal variances was tested using the Brown–Forsythe test. And normal distribution was tested using the Shapiro–Wilk test when the sample size was sufficient. For two group comparisons, the student's *t*‐test was employed for data with normal distribution, while the Mann–Whitney test was used when data were not normally distributed. For multiple group comparisons, the one‐way ANOVA test was conducted followed by either Tukey's Honest Significant Difference Test (HSD) (for between‐group comparisons) or Dunnett's tests (for comparisons to a control group) if the data passed variance equality and normality tests. For data without variance equality and normality, the Kruskal–Wallis test followed by Dunnett's test was performed for multiple comparisons. Two‐tailed statistical significance tests were used throughout. A *p*‐value less than 0.05 was considered statistically significant.

## 3 RESULTS

### 3.1 Single‐cell RNA‐sequencing analysis identifies cell lineages and subtypes in the subacute phase of the TBI

**FIGURE 1:**
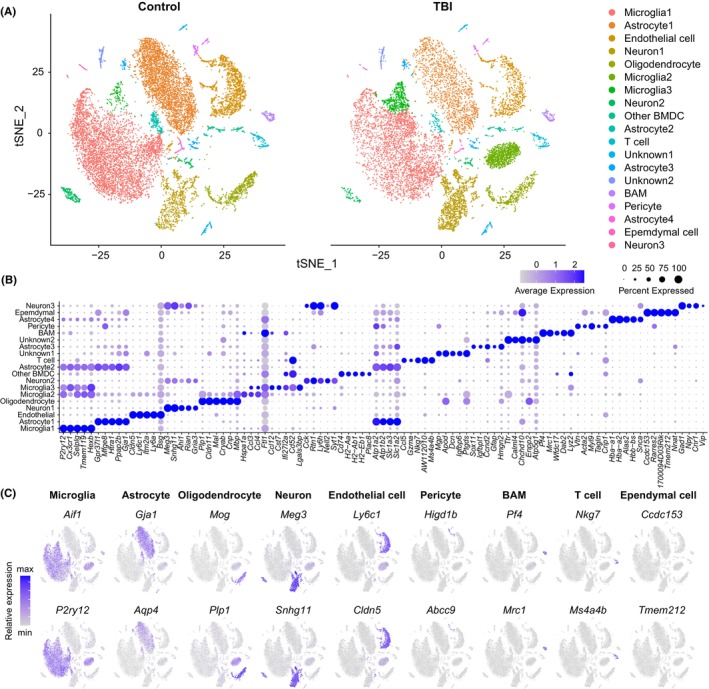
Single‐cell RNA‐sequencing determines major cell types and cell type‐specific genes in the cortex on Day 7 after TBI. (A) t‐SNE plots showing the distribution of different cell clusters in control and TBI groups. Each colored dot represents a cell. (B) Dot plot showing expression of the top five marker genes of each cluster. (C) t‐SNE plots highlighting major cell types by marker gene expression.

To determine cell type‐specific transcriptomic changes in the subacute phase of TBI (7 dpi), we analyzed 14,676 individual cells in the control group and 10,745 cells in the TBI group from a previous study (single‐cell RNA‐sequencing of cortical cells from mice with a fluid percussion injury TBI model).^20^ The high dimensional data were visualized by conducting t‐SNE nonlinear dimensionality reduction to discover distinct clusters of cells (Figure 1A). Nineteen cell clusters were identified and the expression levels of the top five marker genes were analyzed (Figure 1B). According to known cell markers, we identified nine major cell types: microglia (*Aif1*, *P2ry12*), astrocyte (*Gja1*, *Apq4*), oligodendrocyte (*Mog*, *Plp1*), neuron (*Meg3*, *Snhg11*), endothelial cell (*Ly6c1*, *Cldn5*), pericyte (*Higd1b*, *Abcc9*), border‐associated macrophage (*Pf4*, *Mrc1*), T cells (*Nkg7*, *Ms4a4b*), and ependymal cells (*Ccdc153*, *Tmem212*; Figure 1B,C). The two cell clusters whose gene expression signatures did not match any of the known cortical cell types were named unknown1 and unknown2.

### 3.2 Cell–cell interactions and ligand–receptor interaction reconstruction in the subacute phase of TBI

**FIGURE 2:**
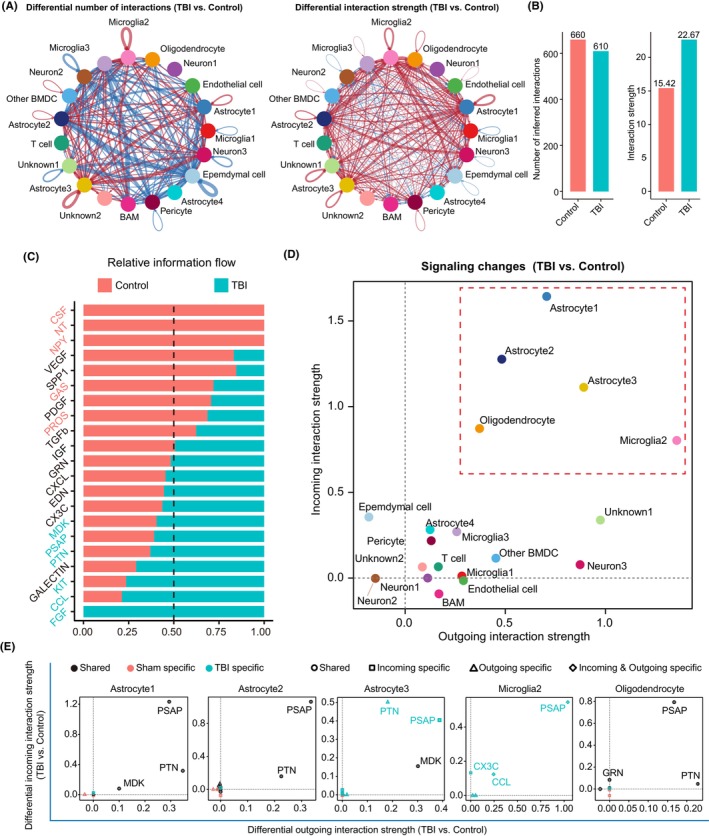
Cell–cell communication network and downstream signaling pathways affected by TBI. (A) Changes in the number and strength of interactions after TBI. Red lines indicate increased number of cell–cell interactions, and the blue lines indicate decreased number of cell–cell interactions. (B) Bar graph showing the differential number and strength of interactions between the control and TBI group. Red lines indicate upregulated cell–cell interaction strength, and the blue lines indicate downregulated cell–cell interaction strength. (C) Relative information flow changes after TBI. (D) The incoming and outgoing interaction strength changes in different clusters of cells after TBI. Cell clusters in the dot frame were further analyzed in (E). (E) The differential incoming and outgoing interactions between control and TBI mice in astrocyte1, astrocyte2, astrocyte3, microglia2, and oligodendrocyte.

Signaling crosstalk via ligand–receptor pairs is critical for cellular physiological and pathological processes. Therefore, we analyzed alterations in cell–cell communications in the subacute phase of TBI using the CellChat R package. The number of inferred cell–cell communications decreased slightly after TBI (Figure 2A,B). However, the strength of cell–cell communication increased markedly after TBI (Figure 2A,B). After TBI, several ligand–receptor interactions were significantly downregulated, including colony‐stimulating factor signaling, neurotrophic (NT) signaling (BDNF‐NTRK2 interaction, Figure S1A), and neuropeptide Y (NPY) signaling (Figure 2C), which generally play a neuroprotective role in TBI or neurodegenerative diseases.^26^, ^27^, ^28^ Additionally, TAM (TYRO3/AXL/MERTK) receptor signaling, including growth arrest‐specific (GAS) signaling and protein S (PROS) signaling, which plays an essential role in immune homeostasis and microglial physiology,^29^, ^30^ was downregulated (Figure 2C). These data suggest that TBI reduces neurotrophic signaling and disrupts immune homeostasis.

Several signaling pathways were significantly enhanced after TBI, including fibroblast growth factor (FGF), CCL, KIT, and non‐canonical neurotrophic factors (MDK, PTN, and PSAP) signaling (Figure 2C). FGF signaling was specifically activated under TBI conditions via FGF9‐FGFR1/2/3 interactions (Figures 2C and S1A,B). FGF9 was released from the neurons and mainly bound to astrocyte receptors (Figure S1B). Activated FGF signaling can suppress the response of astrocytes and accelerate astrocyte deactivation.^31^ CCL signaling was considerably activated after TBI (Figures 2C and S1C), indicating subacute neuroinflammation following TBI. In addition, endothelial cells secreted KIT ligand bound to KIT (Figure S1D) and upregulated KIT signaling in neurons after brain injury (Figures 2C and S1E), which may be involved in promoting neuronal survival and enhancing neurogenesis.^32^, ^33^ Although classical neurotrophic signaling was reduced (Figure S1A), the signaling pathways involving non‐canonical neurotrophic factors MDK, PTN, and PSAP were significantly enhanced after TBI (Figure 2C).

We investigated the cell types in which the cell–cell communication was affected in the subacute phase of TBI. We found that both incoming and outgoing signals from astrocyte1, astrocyte2, astrocyte3, microglia2, and oligodendrocytes were strongly enhanced after TBI (Figure 2D). Notably, signaling was mainly affected in those cells with MDK, PTN, and PSAP signaling, especially astrocytes (Figure 2E). Signaling‐specific cell–cell interaction analysis showed that MDK was exclusively secreted by astrocytes (Figure S2A). PTN was secreted by astrocytes, oligodendrocytes, neurons, pericytes, and endothelial cells (Figure S2B), whereas PSAP was secreted by all cell types (Figure S2C). Notably, enhanced signaling of both PTN and PSAP in astrocyte3 and PSAP in microglia2 was TBI‐specific (Figure 2E). These results indicate that astrocytes and microglia are involved in the pathological process of TBI, at least in the subacute phase, through non‐canonical neurotrophic factor signaling (MDK, PTN, and PSAP).

### 3.3 Time‐dependent expression of PTN, MDK and PSAP after TBI

**FIGURE 3:**
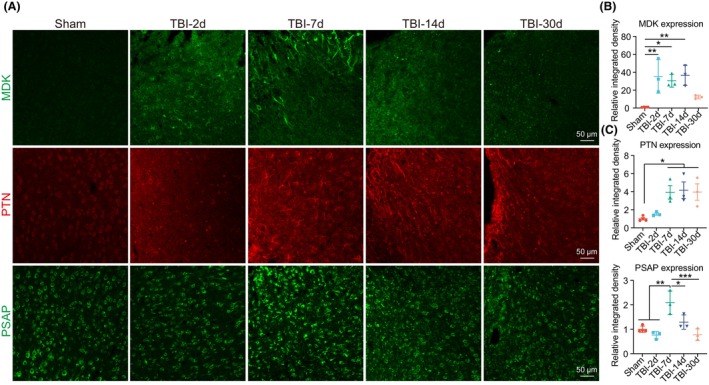
Increased expression of MDK, PTN, and PSAP in the subacute phase of TBI. (A) Representative images showing MDK, PTN, and PSAP expression in the peri‐injured site on Days 2 (TBI‐2d), 7 (TBI‐7d), 14 (TBI‐14d), and 30 (TBI‐30d) after TBI. Scale bar, 50 μm. (B–D) Quantification of fluorescence intensity of MDK (B), PTN (C), and PSAP (D) expression at various time points after TBI (*n* = 4 in the sham group, and *n* = 3 in each TBI group). One‐way ANOVA followed by Tukey's HSD test. *p < 0.05, **p < 0.01.

To validate the upregulation of MDK, PTN, and PSAP signaling after TBI, we measured the expression of MDK, PTN, and PSAP at various time points. After TBI, MDK was significantly upregulated on Days 2, 7, and 14, and then decreased on Day 30 (Figure 3A,B). The upregulation of PTN and PSAP began 7 days after TBI (Figure 3A,C,D). Interestingly, the upregulation of PTN persisted on Days 14 and 30 after TBI (Figure 3A,C), whereas the expression of PSAP decreased to the control level on Days 14 and 30 after TBI (Figure 3A,D). These findings indicate that the expression of MDK, PTN, and PSAP was mainly upregulated in the subacute phase of TBI.

**FIGURE 4:**
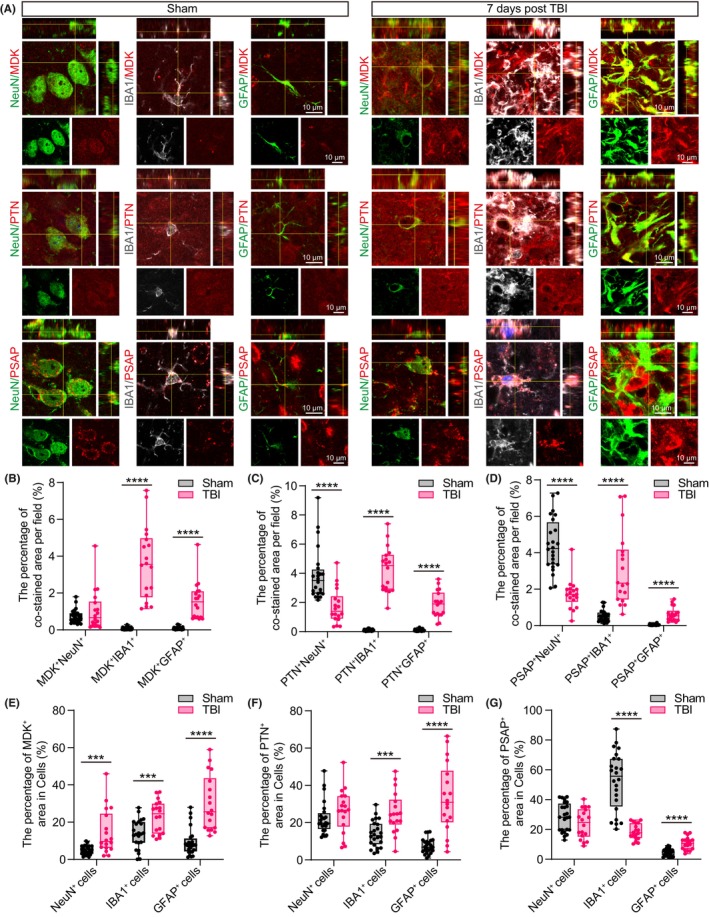
MDK, PTN, and PSAP expressions in major neural cell types after TBI. (A) Double immunostaining showing MDK, PTN, and PSAP expression in the neuron (NeuN), microglia (IBA1), and astrocyte (GFAP) on Day 7 after TBI. Scale bar, 10 μm. (B–D) Quantification of the co‐stained area of MDK (B), PTN (C), and PSAP (D) staining with NeuN, IBA1, and GFAP staining in the peri‐injured site of the brain on Day 7 after TBI. (E–G) Quantification of the MDK‐ (E), PTN‐ (F), and PSAP‐positive (G) area in the neuron (NeuN), microglia (IBA1), and astrocytes (GFAP) in the peri‐injured site of the brain on Day 7 after TBI. The Student's *t‐*test was performed. Mann–Whitney test was used when the sample distributions were skewed. Six fields per mouse were imaged, a total of 24 fields in the sham group and 18 fields in the TBI group. ***p < 0.001, ****p < 0.0001.

We then investigated the cell types that were involved in the upregulation of MDK, PTN, and PSAP expression and found that they were mostly expressed in the neurons of sham mice and were weakly expressed in microglia and astrocytes (Figure 4A). We quantified the expression of MDK, PTN, and PSAP in the major neural cell types: neurons (NeuN), microglia (IBA1), and astrocytes (GFAP) in the subacute phase of TBI (7 days after TBI). Compared with that in the sham mice, the MDK^+^NeuN^+^ colocalization area did not change, whereas the PTN^+^NeuN^+^ and PSAP^+^NeuN^+^ areas were significantly reduced in TBI‐7d mice (Figure 4B–D). Positive areas of MDK, PTN, and PSAP co‐labeled with IBA1 and GFAP remarkably increased after TBI, indicating that astrocytes and microglia are the major sources of increased expression of non‐canonical neurotrophic factors (Figure 4B–D). In light of the TBI‐induced neuronal loss and glial activation (Figure S3A–D), we also analyzed MDK, PTN, and PSAP expression levels per cell by normalizing them to the area of each cell type. MDK expression in neurons (area of MDK^+^NeuN^+^/area of NeuN^+^) increased, whereas PTN and PSAP expression remained unchanged (Figure 4E–G), suggesting that the decreased PTN^+^NeuN^+^ and PSAP^+^NeuN^+^ areas after TBI were due to neuronal loss. In both microglia and astrocytes, the expression of MDK and PTN was significantly upregulated (Figure 4E,F). PSAP expression increased in astrocytes but decreased in microglia after normalization to the area of IBA1 (Figure 4G), which could partially be attributed to microglial activation‐induced hypertrophy (amoeboid morphology). PSAP is a lysosomal protein that regulates lysosomal function.^34^ Microglia phagocytose dead cells and myelin debris after brain injury,^35^ which may exhaust functional lysosomes and induce a reduction in PSAP expression in microglia. These findings were partly consistent with our cell–cell communication results showing that MDK, PTN, and PSAP signaling was upregulated in the astrocytes (Figure 2E). Overall, the expression of the non‐canonical neurotrophic factors MDK, PTN, and PSAP was enhanced in the subacute phase of TBI, mainly because of their increased expression in astrocytes and microglia.

We also examined the expression of MDK, PTN, and PSAP in the plasma of patients with TBI. Plasma was collected from five people with no incidence of TBI (controls) and 14 patients with TBI (10 with mild TBI and four with moderate/severe TBI treated with decompressive craniectomy), and MDK, PTN, and PSAP expression was detected using dot blotting. Notably, correlation analysis revealed positive correlations among the expression of MDK, PTN, and PSAP (Figure S4A–D), especially between MDK and PTN, indicating that MDK, PTN, and PSAP exhibit a similar expression pattern after TBI. However, the relative expression of MDK, PTN, and PSAP did not increase in both these cases: 1 day after mild TBI and 7 days after moderate/severe TBI (Figure S4A,E). This observation may be attributed to the limited sample size and time points of the examination.

### 3.4 Inflammation upregulates PTN, MDK and PSAP expression in astrocyte

**FIGURE 5:**
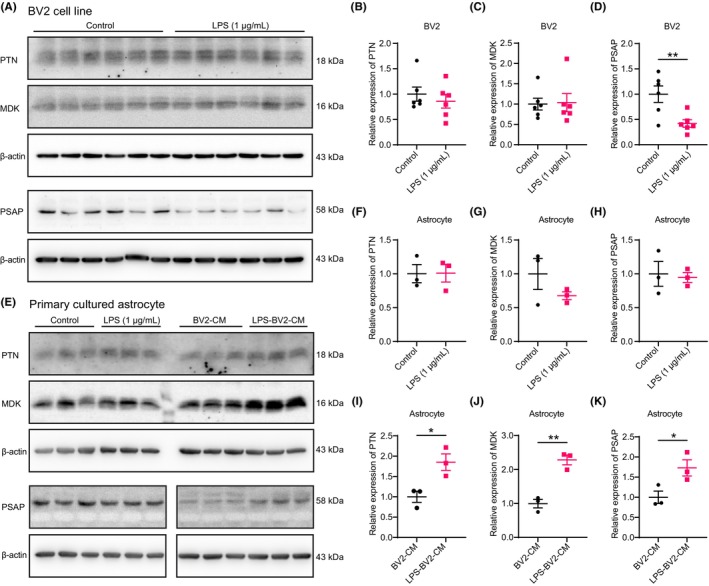
Inflammatory condition upregulates MDK, PTN, and PSAP expression in the astrocytes. (A–D) Representative immunoblots (A) and quantification (B–D) showing expression of MDK, PTN, and PSAP in BV2 cells after LPS stimulation. (E) Representative images showing the MDK, PTN, and PSAP expression in astrocytes after treatment with the conditioned medium from LPS‐stimulated BV2 cells. (F–H) Bar graphs show that LPS stimulation did not affect MDK, PTN, and PSAP expression in the astrocytes. (I–K) Bar graphs showing that the conditioned medium from LPS‐stimulated BV2 cells upregulated MDK, PTN, and PSAP expression in the astrocytes. The Student's *t‐*test was performed, while the Mann–Whitney test was used when the sample distributions were skewed. *p < 0.05, **p < 0.01.

We examined the expression of MDK, PTN, and PSAP in microglia and astrocytes under inflammatory conditions (LPS stimulation) in vitro. Our results revealed that LPS stimulation did not affect MDK and PTN expression in BV2 microglia (Figure 5A–C) or in primary cultured astrocytes (Figure 5E–H). However, PSAP expression was significantly decreased in BV2 cells but not in astrocytes (Figure 5A,D), consistent with the in vivo findings (Figure 4G). These data suggest that the upregulation of MDK, PTN, and PSAP signaling is not directly mediated by LPS‐induced inflammatory responses. This result was partly consistent with the in vivo findings that PTN and PSAP expression was upregulated on Day 7 after TBI rather than during the earlier stage of TBI (2 days after TBI), indicating that secondary injury plays a potential role in PTN and PSAP expression. To validate this hypothesis, we treated astrocytes with conditioned medium collected from LPS‐stimulated BV2 cells and observed considerably higher levels of MDK, PTN, and PSAP expression in astrocytes (Figure 5E,I–K). These findings suggest that the inflammatory response is involved in the upregulation of MDK, PTN, and PSAP expression in astrocytes.

### 3.5 PTN, MDK and PSAP promote NPC proliferation and neurite growth

**FIGURE 6:**
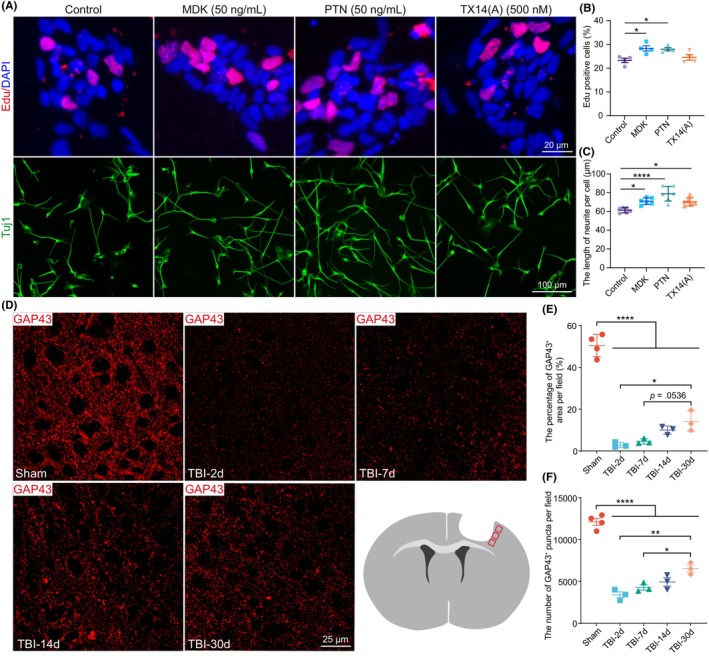
The roles of the MDK, PTN, and PSAP in NSC proliferation and neurite growth. (A) Representative images showing the NSC proliferation and neurite growth after MDK, PTN, and PSAP treatment. Scale bar, 20 μm and 100 μm, respectively. (B) Bar graph showing the increased number of EdU‐labeled NSCs after MDK and PTN treatment but not PSAP treatment (*n* = 4 in each group). (C) Bar graph showing that the total length of neurite per cell was significantly increased after MDK, PTN, and PSAP treatment (*n* = 6 in each group). One‐way ANOVA followed by Dunnett's tests for data in B and C. (D) Representative images showing GAP43 expression and a schematic illustration of the imaging area (indicated by the red boxes). (E and F) Bar graph showing that the percentage of GAP43‐positive area (E) and the number of GAP43‐positive puncta (F) were decreased after TBI and exhibited spontaneous recovery over time. One‐way ANOVA followed by Tukey's HSD test. *p < 0.05, **p < 0.01, ****p < 0.0001.

Neurotrophic factors are implicated in neural regeneration following brain injury. To test whether MDK, PTN, and PSAP regulate the proliferation of NPCs, we treated human iPSC‐derived NPCs with recombinant MDK, PTN, and the PSAP‐derived peptide TX14(A). The EdU cell proliferation assay indicated that NPC proliferation was significantly increased by MDK and PTN treatment (Figure 6A–C). We also examined the roles of MDK, PTN, and PSAP in neurite outgrowth. Recombinant proteins and PSAP‐derived peptides were individually applied to human iPSC‐derived neurons, which were then cultured for 72 h. The length of Tuj1‐positive neurite was significantly increased by treatment with MDK, PTN, or TX14(A). These findings indicate that MDK, PTN, and PSAP play potential roles in neural regeneration.

We then explored neural regeneration in the peri‐injured cortical area using growth‐associated protein 43 (GAP43) immunostaining. Both the percentage of GAP43‐positive area and the number of GAP43‐positive puncta significantly decreased after TBI compared with that in the sham group, and this trend persisted even 30 days post‐TBI (Figure 6D–F). It is worth noting that both the percentage of GAP43‐positive area and the number of GAP43‐positive puncta increased in a time‐dependent manner and showed significant upregulation at 30 days post‐TBI (*p* = 0.0536 for the GAP43‐positive area) when compared to the results at 2 and 7 days post‐TBI (Figure 6E,F). In combination with the in vitro findings, we inferred that the upregulation of MDK, PTN, and PSAP expression in the subacute phase of TBI is associated with spontaneous neural regeneration after TBI.

### 3.6 PTN, MDK and PSAP have a limited role in inflammatory response regulation

MDK, PTN, and PSAP modulate inflammatory responses in various diseases.^36^, ^37^ To explore the role of MDK, PTN, and PSAP in immunomodulation, BV2 cells were directly stimulated with MDK, PTN, and TX14(A). Twenty‐four hours later, we measured the expression of inflammation‐associated genes, including *Il1b*, *Il4*, *Il6*, *Il10*, *Il13*, *Ccl2*, *Tnfa*, *Tgfb*, *Nos2*, *Cd86*, *Cd206*, and *Arg1*. The results showed that PTN and TX14(A) upregulated *Il1b* expression in BV2 cells (Figure S5A). TX14(A) also induced *Il10* and *Tgfb* expression (Figure S5A). We investigated whether MDK, PTN, and PSAP could regulate the LPS‐induced inflammatory response. *Il1b*, *Il6*, *Il13*, *Ccl2*, *Tnfa*, *and Nos2* mRNA expression was significantly upregulated after LPS stimulation in BV2 cells (Figure S5B). However, MDK, PTN, and TX14(A) did not affect the expression of LPS‐induced inflammation‐associated genes (Figure S5B). These findings suggested that MDK, PTN, and PSAP play limited roles in inflammatory regulation.

## 4 DISCUSSION

High‐throughput single‐cell RNA‐sequencing enables us to uncover novel cell subtypes, cell–cell interactions, and signaling pathways under pathological conditions, providing new insights for developing therapeutic strategies. In the present study, using high‐throughput single‐cell RNA‐sequencing analysis, we unveiled damage‐associated cell–cell communication changes in the subacute phase of TBI. Our results demonstrated that the canonical neurotrophic factor signaling (NT and NPY) was significantly downregulated during the subacute phase of TBI. However, the signaling of the non‐canonical neurotrophic factors MDK, PTN, and PSAP was enhanced in the subacute phase of TBI. The in vivo results showed that the expression of MDK, PTN, and PSAP after TBI was time‐dependent. An in vitro study revealed that MDK, PTN, and PSAP expression was upregulated in astrocytes after inflammatory stimulation, which may play a role in neural regeneration rather than in inflammation regulation.

Single‐cell RNA‐sequencing enables the inference of cell–cell communication by analyzing the expression of genes encoding the corresponding ligands, receptors, and intermediary proteins across the interacting cell populations. In this study, we chose the datasets of the control and TBI groups from the reported single‐cell RNA‐sequencing data to explore the common pathological changes in the subacute phase of TBI. We found that the number of cell–cell interactions decreased, while the strength of cell–cell interactions increased, indicating that TBI disrupts neural homeostasis. Information flow analysis, which quantified the total communication probability within the inferred network, revealed that FGF (FGF9‐FGFR1/2/3 interactions) and CCL signaling were upregulated after TBI. These two signaling pathways were mainly derived from the cell–cell interactions among glial cells and inflammatory cells (Figure S1B,C), which is supported by increased glial activation and neuroinflammation in TBI.^38^ The cell–cell communication analysis also revealed that microglia, astrocytes, oligodendrocytes, and some bone marrow‐derived inflammatory cells were the most affected cell types in the subacute phase of TBI. Detailed ligand–receptor interaction analysis of these cell types demonstrated that canonical neurotrophic factor (BDNF and NPY)‐related signaling was downregulated. These results are consistent with those of previous studies that have reported reduced BDNF expression in the brain after injury^39^, ^40^ and the loss of NPY‐positive cells in the hippocampus in the experimental model of TBI.^41^ Both BDNF and NPY are involved in neuroprotection and neurogenesis in neurological diseases^28^, ^42^ and downregulating BDNF and NPY signaling may be detrimental to spontaneous recovery after TBI. Interestingly, our findings revealed that non‐canonical neurotrophic signaling (MDK, PTN, and PSAP) was enhanced after TBI. Notably, PTN and PSAP signaling was specifically upregulated following TBI in astrocyte3 and microglia2 cells, suggesting that these proteins play distinct roles in these cell types.

MDK and PTN, the heparin‐binding neurite outgrowth‐promoting factors, have similar structures and functions and have been verified to belong to the neurotrophic factor family.^43^ They regulate neural development and neural stem cell migration and proliferation.^43^, ^44^ Upregulated MDK and PTN expression has been found following CNS injury and neurodegenerative diseases, including ischemic stroke, Alzheimer's disease, and multiple system atrophy.^43^, ^44^ In the rodent TBI model, we found that both MDK and PTN expression increased after TBI. However, the time‐dependent expression patterns of MDK and PTN differed. Upregulation of MDK expression occurred earlier than that of PTN after TBI, but PTN expression persisted longer than MDK expression, suggesting that MDK and PTN play different roles in TBI. MDK and PTN were mainly expressed in the neurons and were markedly upregulated in the astrocytes after TBI. This finding is consistent with that of an in vitro study, which indicated that MDK and PTN expression significantly increased in primary cultured astrocytes after the treatment with LPS‐stimulated BV2‐derived conditioned medium. However, direct LPS stimulation did not increase the MDK and PTN expression in astrocytes, indicating the potential role of inflammatory cytokines in regulating MDK and PTN expression in astrocytes.

PSAP is a nonenzymic glycoprotein that is targeted at lysosomes.^45^ PSAP can be secreted into the extracellular space under conditions of stress and injury to prevent neuronal and glial injury.^46^ In the present study, we found that PSAP expression was temporarily increased 7 days after TBI. Microglia were the main cell type with elevated PSAP expression after TBI, indicating that PSAP may be associated with microglial activation.^47^ However, an in vitro study showed that LPS stimulation decreased PSAP expression in BV2 cells. Perhaps, microglia phagocytose dead cells or myelin debris promotes PSAP accumulation in lysosomes or exhaust PSAP.

The roles of MDK, PTN, and PSAP in TBI are not fully understood. Both MDK and PTN play an anti‐apoptotic role by inhibiting caspase‐3 expression.^48^, ^49^ PSAP can promote neuron survival by inhibiting ferroptosis under chronic oxidative stress.^50^ Upregulation of MDK, PTN, and PSAP expression after TBI may play a glioprotective and neuroprotective role.^43^, ^46^, ^51^ Spontaneous synaptogenesis and axonal sprouting are involved in spontaneous recovery after brain injury,^52^ which may depend on neurotrophic factors.^53^ Our result demonstrated that the canonical neurotrophic factor signaling pathway was downregulated in the subacute phase of TBI, while the non‐canonical neurotrophic signaling pathway was upregulated, suggesting that the non‐canonical neurotrophic factors are involved in spontaneous neural repair after TBI. This hypothesis was supported by an in vitro study, which revealed that MDK and PTN promoted iPSC‐derived NPCs proliferation and that MDK, PTN, and PSAP‐derived peptides enhanced neurite growth of iPSC‐derived neurons.

MDK, PTN, and PSAP can also mediate inflammation under pathological conditions.^37^, ^54^ MDK deficiency reduces M1 phenotype microglia/macrophage infiltration after TBI.^55^ Overexpression of PTN in mice significantly enhances LPS‐induced inflammatory cytokine expression.^56^, ^57^ Transplantation of PSAP‐deficient bone marrow into low‐density lipoprotein receptor knockout mice decreases plaque inflammation in atherosclerosis.^37^ These studies suggest that MDK, PTN, and PSAP play a pro‐inflammatory role. Our in vitro study demonstrated that MDK, PTN, and PSAP play limited roles in inflammatory regulation with/without LPS stimulation. Delayed upregulation of PTN and PSAP expression after TBI may also imply their limited role in neuroinflammation, at least during the subacute phase of TBI.

Single‐cell RNA‐sequencing analysis revealed the comprehensive cell–cell interactions in the subacute phase of TBI. Taking advantage of the cell–cell communication analysis, we discovered that the signaling of the non‐canonical neurotrophic factors MDK, PTN, and PSAP was enhanced compared with the signaling of the canonical neurotrophic factors BDNF and NPY, after TBI. The upregulation of MDK, PTN, and PSAP expression after TBI may play a role in spontaneous neurogenesis and axonal regeneration after TBI. These new insights can help understand the complex pathological processes of TBI and provide therapeutic targets for TBI. Notably, the TBI model used in the present study was a weight loss‐induced TBI model, which is a more severe model than the fluid percussion injury TBI model used in single‐cell RNA‐sequencing studies. Consistent results from the two different TBI models suggest that enhanced signaling of MDK, PTN, and PSAP is a common pathological feature of both mild and severe TBI. However, the potential of exogenous MDK, PTN, and PSAP to promote brain repair and the underlying mechanisms behind it remain unclear, and further studies in an experimental model of TBI are required.

## 5 CONCLUSIONS

In summary, we demonstrated that the non‐canonical neurotrophic factors MDK, PTN, and PSAP were upregulated in the subacute phase of TBI and revealed their roles in promoting neuroregeneration. Our findings provide new insights into TBI pathology and offer potential therapeutic targets for TBI intervention.

## AUTHOR CONTRIBUTIONS

X.Q. carried out experiments, analyzed data, and prepared the manuscript. Y.G. performed the immunofluorescence staining and statistical analysis. M.F.L. calculated a Glasgow Coma Scale (GCS) score of TBI participants and collected blood samples. B.Z. assisted in the qPCR study. J.L. and J.F.W. contributed to the design of in vitro study and the manuscript revision process. M.L. and X.Q. conceived the study. M.L. supervised the experiment and revised the manuscript.

## CONFLICT OF INTEREST STATEMENT

The authors declare no conflict of interest.

## CONSENT FOR PUBLICATION

Not applicable.

## Supporting information


Table S1.

Table S2.

Table S3.

Data S1.

Figure S1.

Figure S2.

Figure S3.

Figure S4.

Figure S5.

## Data Availability

The data that support the findings of this study are available in Gene Expression Omnibus at https://www.ncbi.nlm.nih.gov/geo/, reference number GSE160763. These data were derived from the following resources available in the public domain: ‐ Single‐cell sequencing of cortical cells following murine tr, https://www.ncbi.nlm.nih.gov/geo/query/acc.cgi?acc=GSE160763.
