# Supplementary material for: Single‐cell RNA‐sequencing analysis reveals enhanced non‐canonical neurotrophic factor signaling in the subacute phase of traumatic brain injury
Source: CNS Neurosci Ther. 2023 Jun 2;29(11):3446–59. doi: 10.1111/cns.14278 (PMC10580338; doi:10.1111/cns.14278)
Supplement: Supplementary file 4 — Data S1. [file CNS-29-3446-s009.docx]

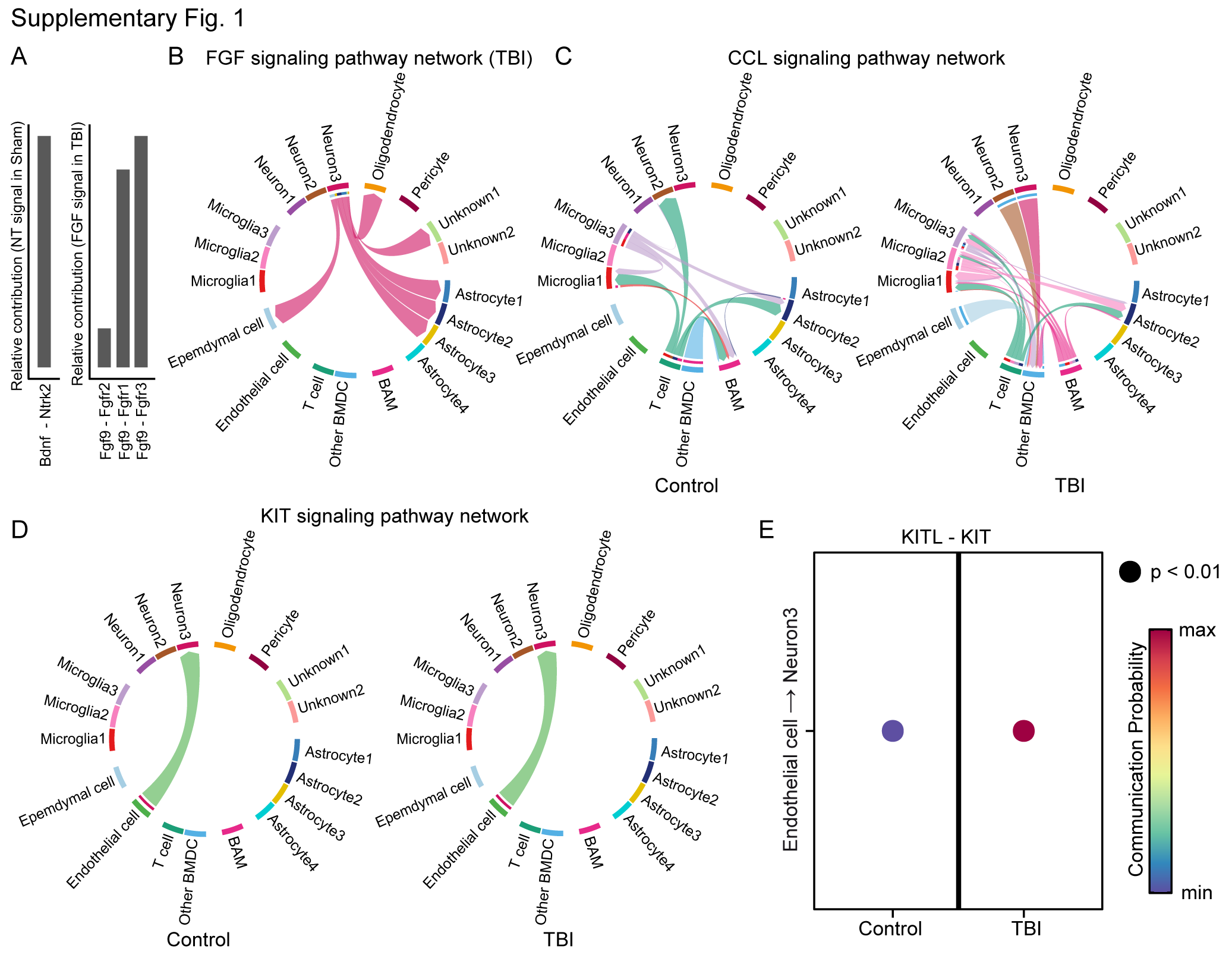


Supplementary FIGURE 1 Cell­­–cell communication changes in the NT, FGF, CCL, and KIT signaling pathways after TBI. (A) The relative contribution of ligand-receptor interaction in the NT and FGF signaling. (B) The FGF signaling pathway network is a TBI-specific pathway. (C) The CCL signaling pathway network in the control and TBI group. (D) The KIT signaling pathway network in the control and TBI group. (E) The communication probability between endothelial cells and neurons in the control and TBI group.


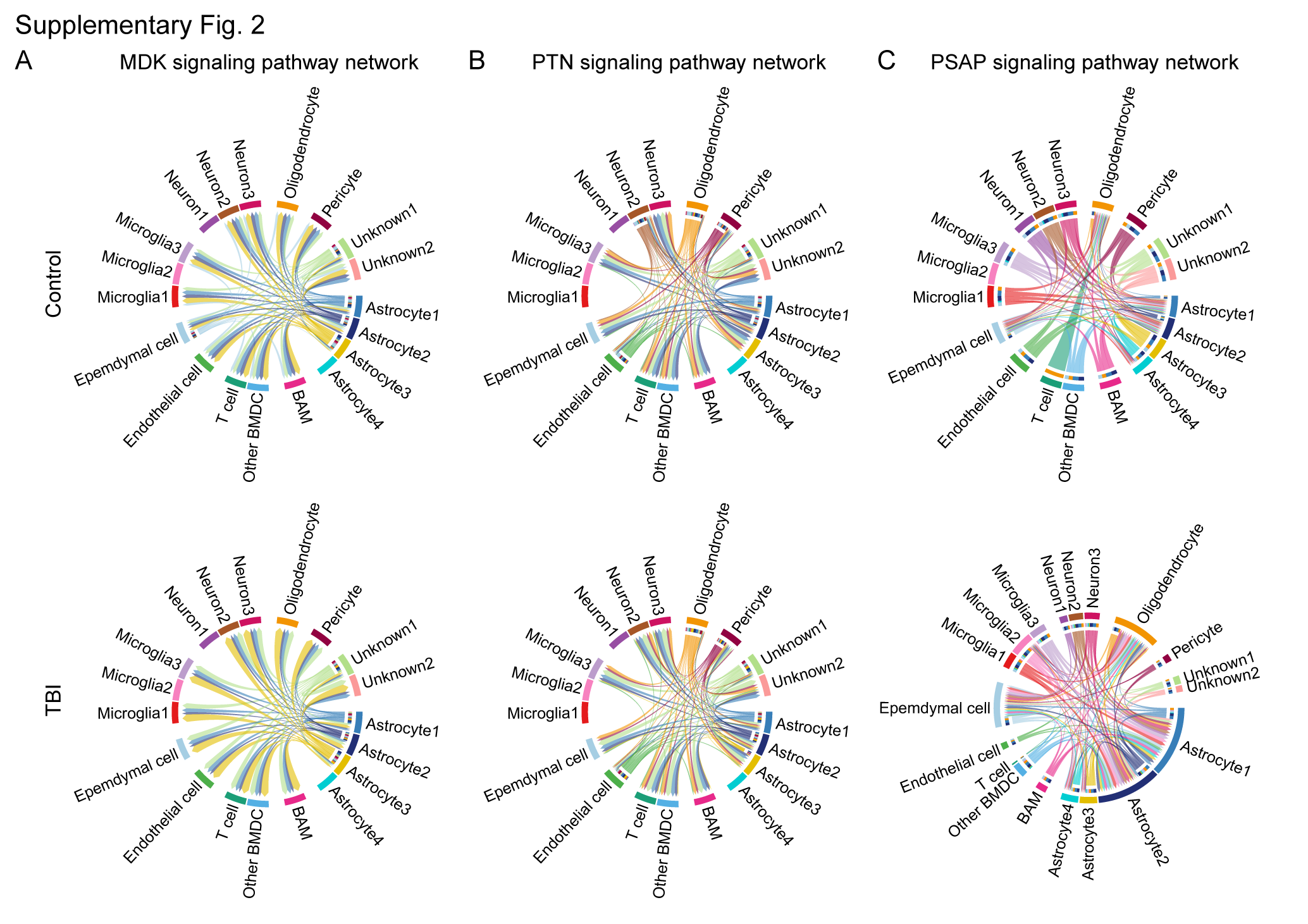


**Supplementary FIGURE 2** Cell–cell communication changes of the MDK, PTN, and PSAP signaling pathways after TBI. (A) The MDK signaling pathway network changes after TBI. (B) The PTN signaling pathway network changes after TBI. (C) The PSAP signaling pathway network changes after TBI.


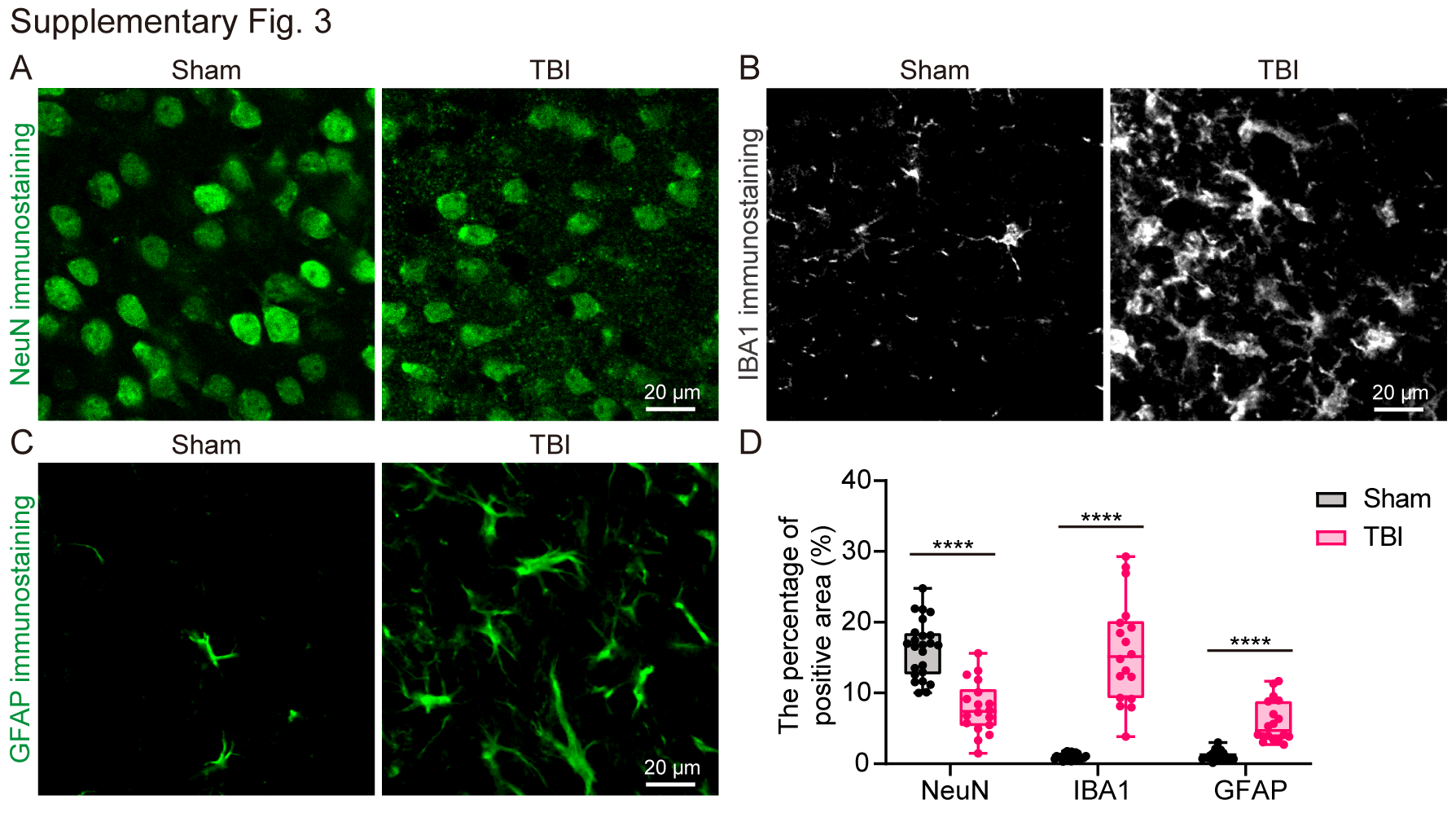


**Supplementary FIGURE 3** NeuN, IBA1, and GFAP expression after brain injury. (A­­–C) Representative images showing the NeuN (A), IBA1 (B), and GFAP (C) expression in the peri-injured site at 7 days after TBI. Scale bar, 20 μm. (D) Quantification of the NeuN, IBA1, and GFAP positive area in the peri-injured site of the brain at 7 days after TBI. The Student’s t-test was performed, while the Mann–Whitney test was used when the sample distributions were skewed. Six fields per mouse were imaged, a total of 24 fields in the sham group and 18 fields in the TBI group. *****p* < 0.0001.


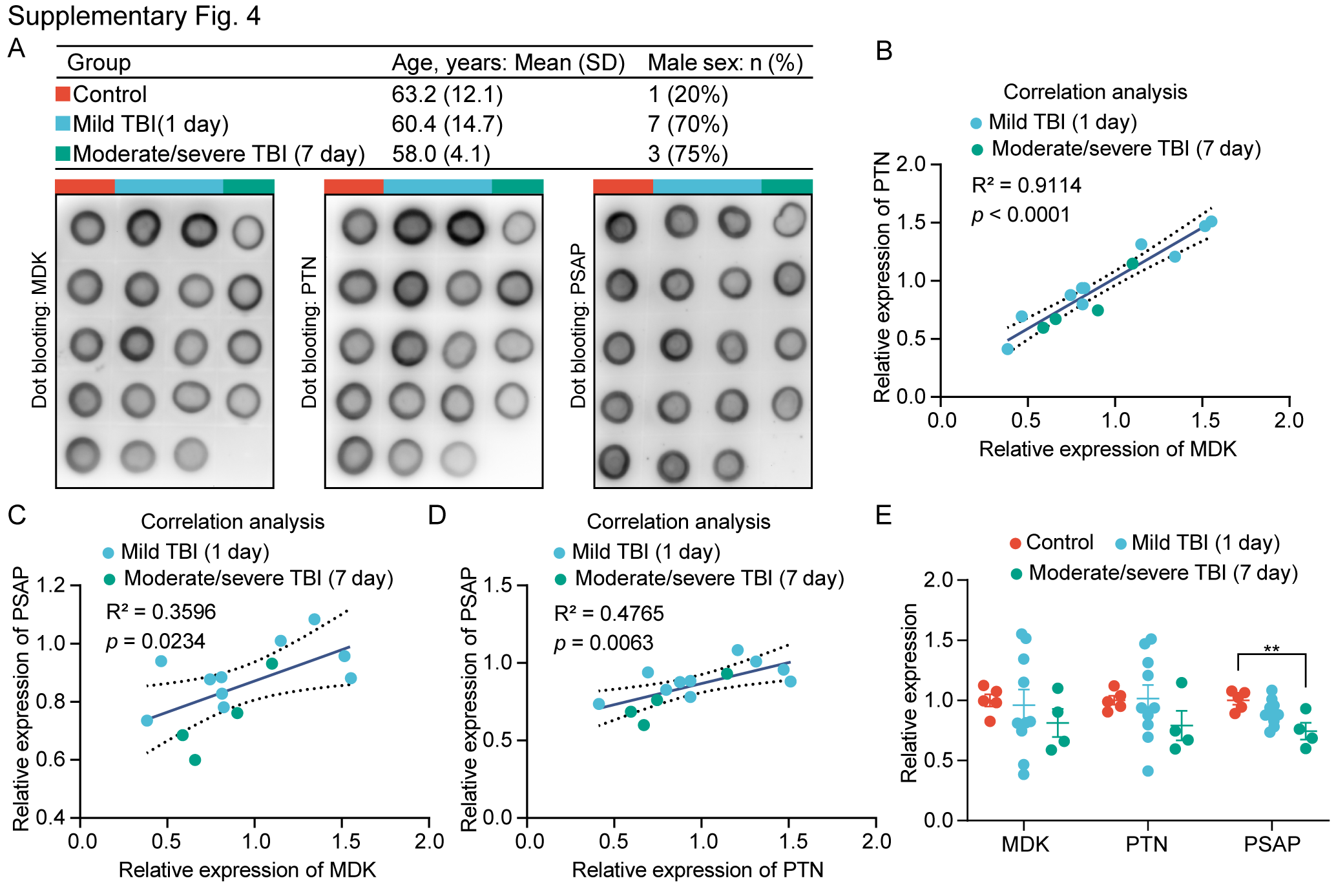


Supplementary FIGURE 4 Positive correlation of MDK, PTN, and PSAP expression in plasma from TBI patients. (A) The patients’ general information and the dot blotting showing the expression of MDK, PTN, and PSAP in the plasma of TBI patients. The patients with TBI were divided into two groups according to a Glasgow Coma Scale (GCS) score: mild TBI (GCS ≥13) and moderate/severe TBI (GCS < 13). Two microliter plasma was dropped onto an NC membrane and air-dried. The NC membrane was rinsed with TBST and blocked with a 5% non-fat milk TBST solution. The membrane was incubated with corresponding primary antibodies and imaged after corresponding secondary antibody incubation. (B­­–D) The correlation analysis among MDK, PTN, and PSAP expression. Positive correlations were observed among the relative expression of MDK, PTN, and PSAP in the plasma of TBI patients. (E) The statistical analysis of the MDK, PTN, and PSAP expression in the plasma of TBI patients. There were no significant differences in MDK and PTN expression among control, mild TBI (1 day), and moderate/severe TBI (7 day). The expression of PSAP was significantly decreased in the moderate/severe TBI (7 day) group compared with the control group. One-way ANOVA test followed by the Dunnett’s tests was conducted. **p < 0.01.


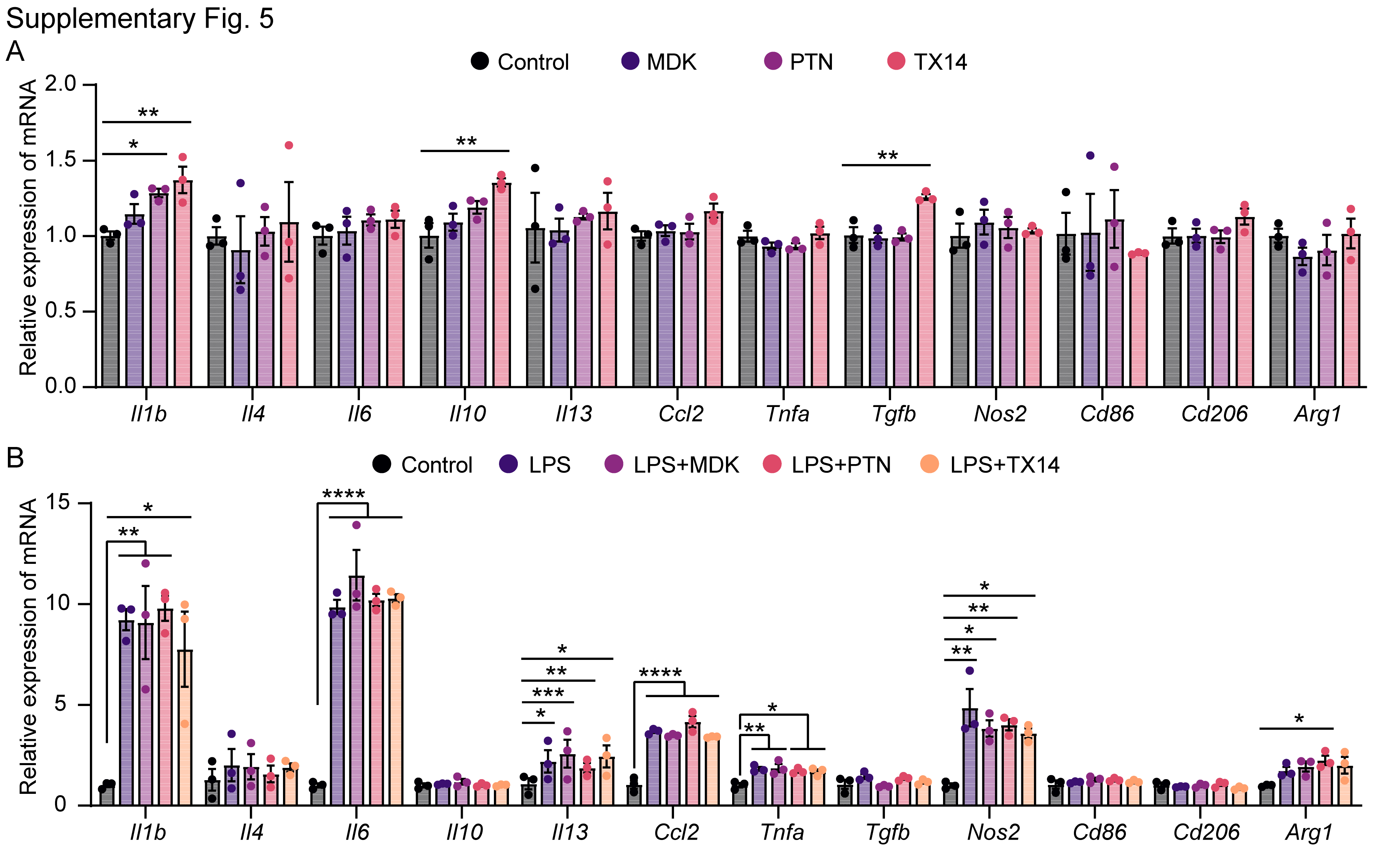


Supplementary FIGURE 5 Limited effects of MDK, PTN, and PSAP in inflammatory regulation. (A) Bar graphs showing the direct role of MDK, PTN, and PSAP in inflammatory gene expression in BV2 cells (n = 3 in each group). (B) Bar graphs showing the role of MDK, PTN, and PSAP in regulating inflammatory gene expression in LPS-stimulated BV2 cells (n = 3 in each group). One-way ANOVA test followed by the Dunnett’s tests was performed. **p* < 0.05, ***p* < 0.01, ****p* < 0.001, *****p* < 0.0001.
