# Supplementary figures and images for: Single‐cell RNA‐sequencing analysis reveals enhanced non‐canonical neurotrophic factor signaling in the subacute phase of traumatic brain injury
Source: CNS Neurosci Ther. 2023 Jun 2;29(11):3446–59. doi: 10.1111/cns.14278 (PMC10580338; doi:10.1111/cns.14278)

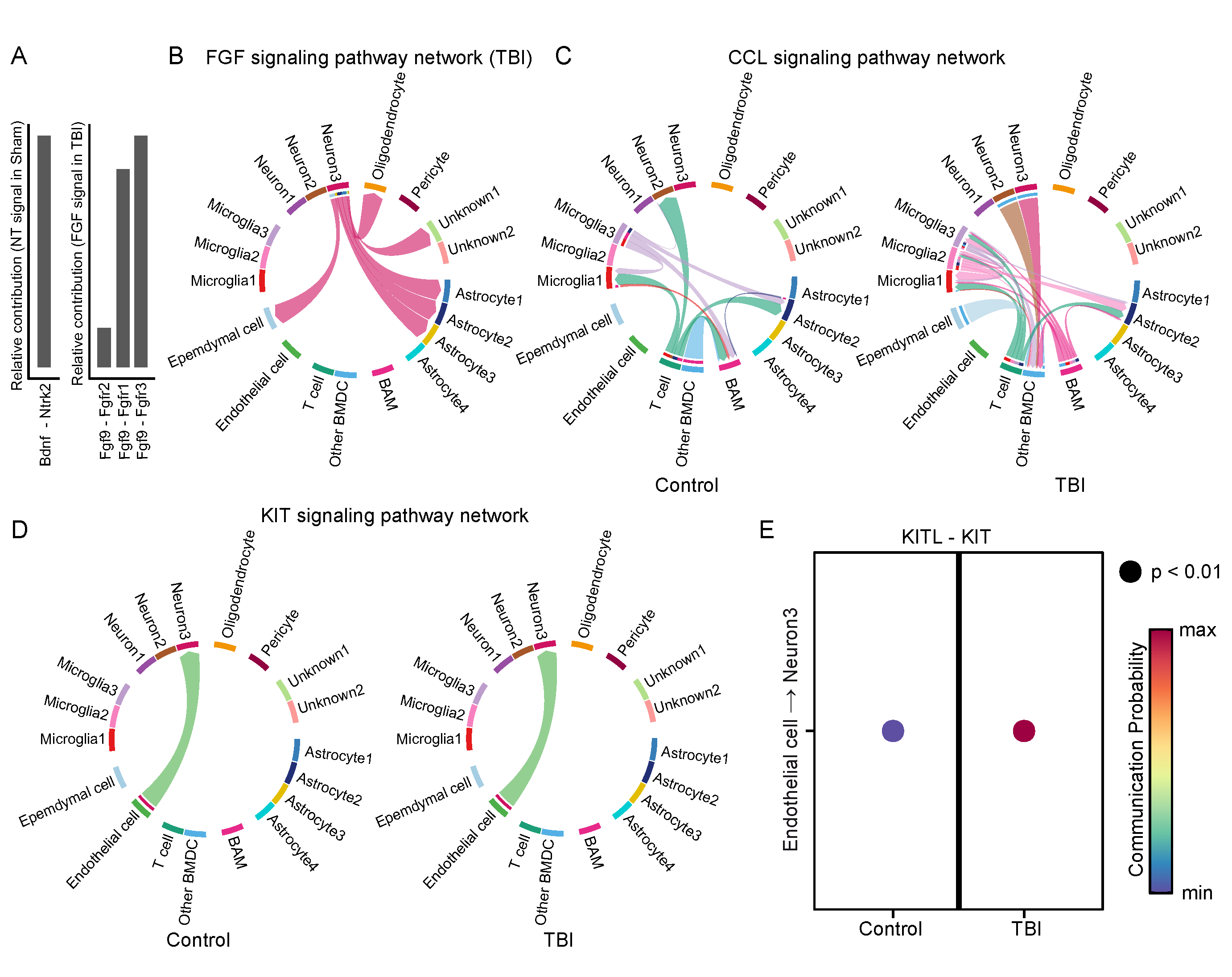

Supplement: Supplementary file 5 — Figure S1. [file CNS-29-3446-s005.tiff]

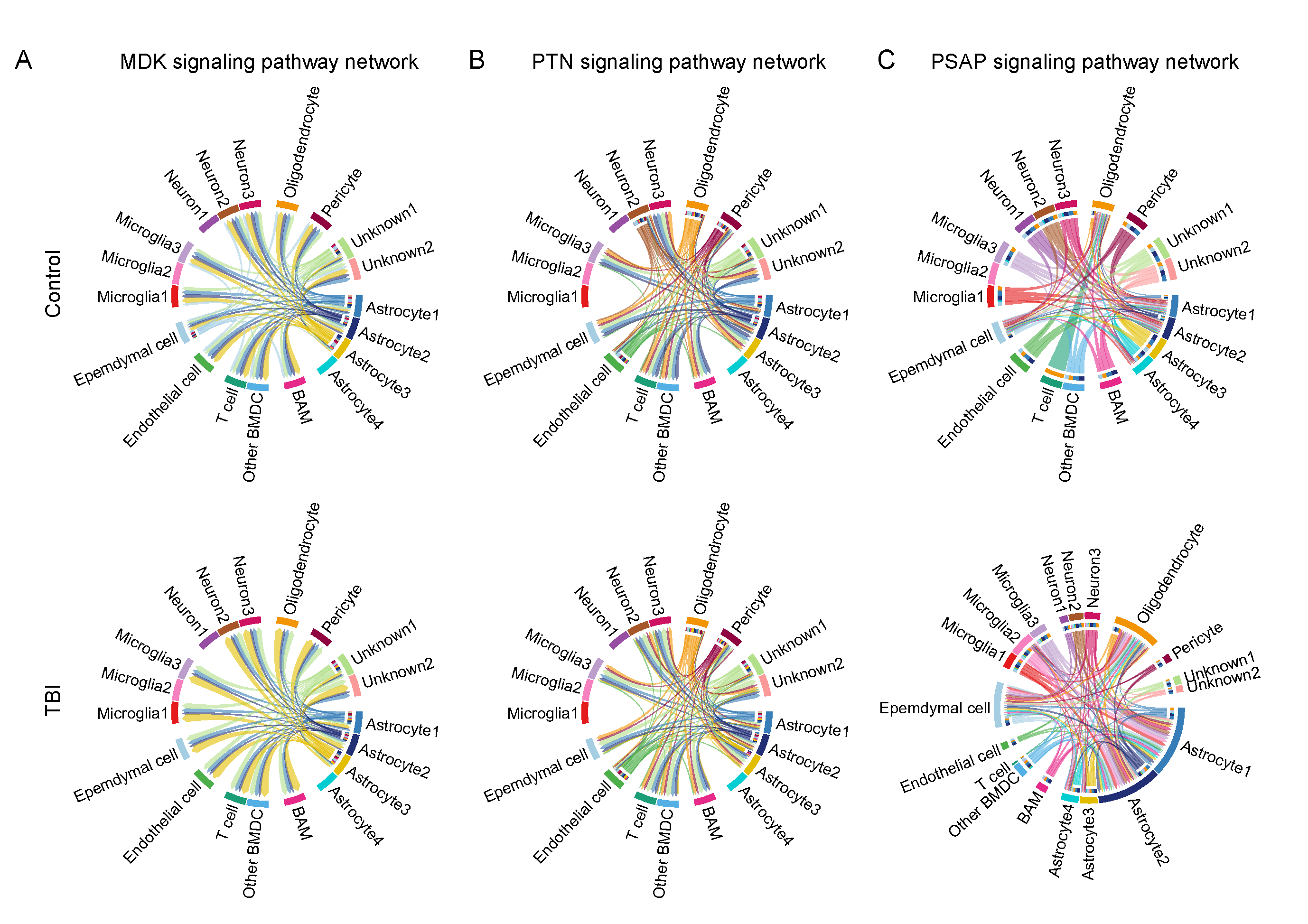

Supplement: Supplementary file 6 — Figure S2. [file CNS-29-3446-s008.tiff]

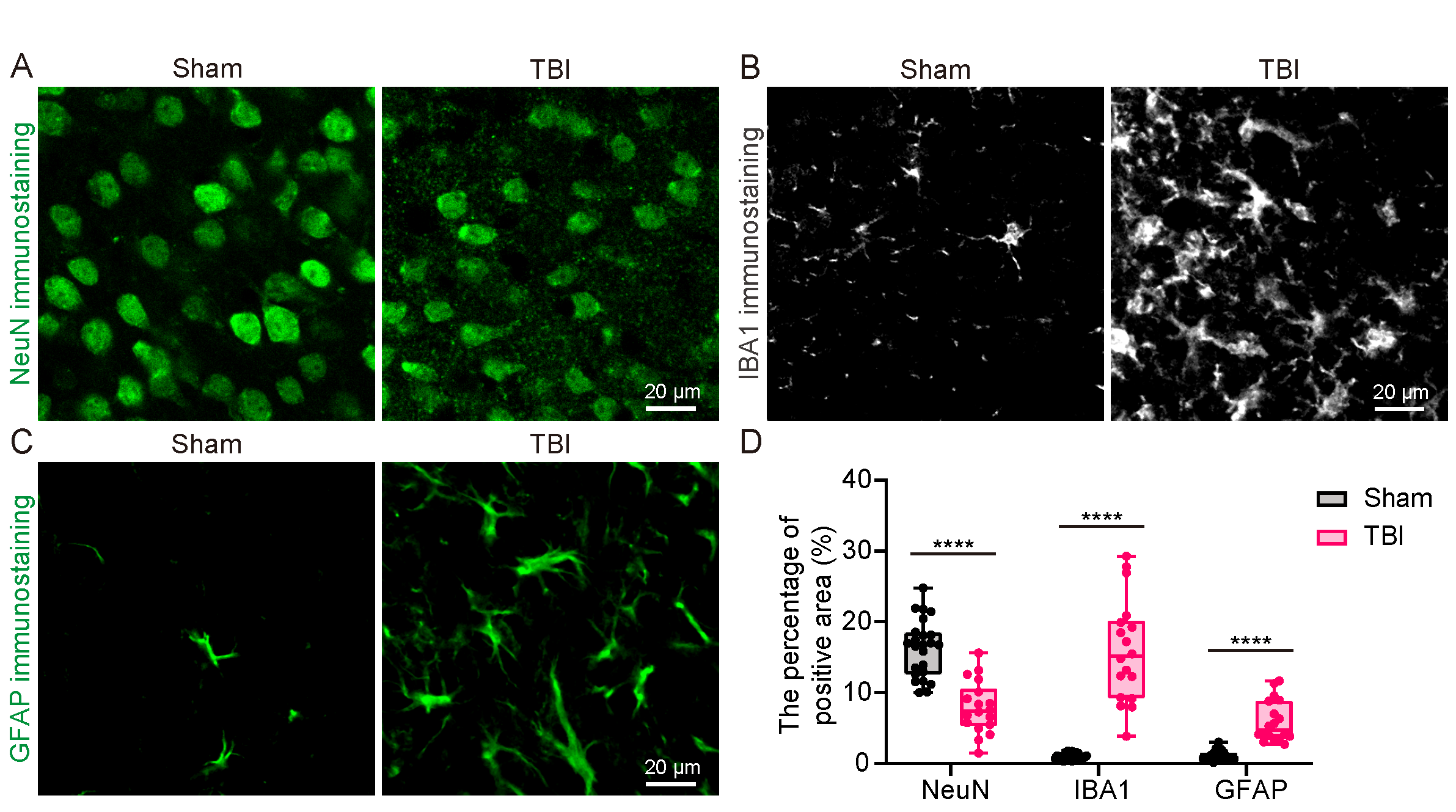

Supplement: Supplementary file 7 — Figure S3. [file CNS-29-3446-s002.tiff]

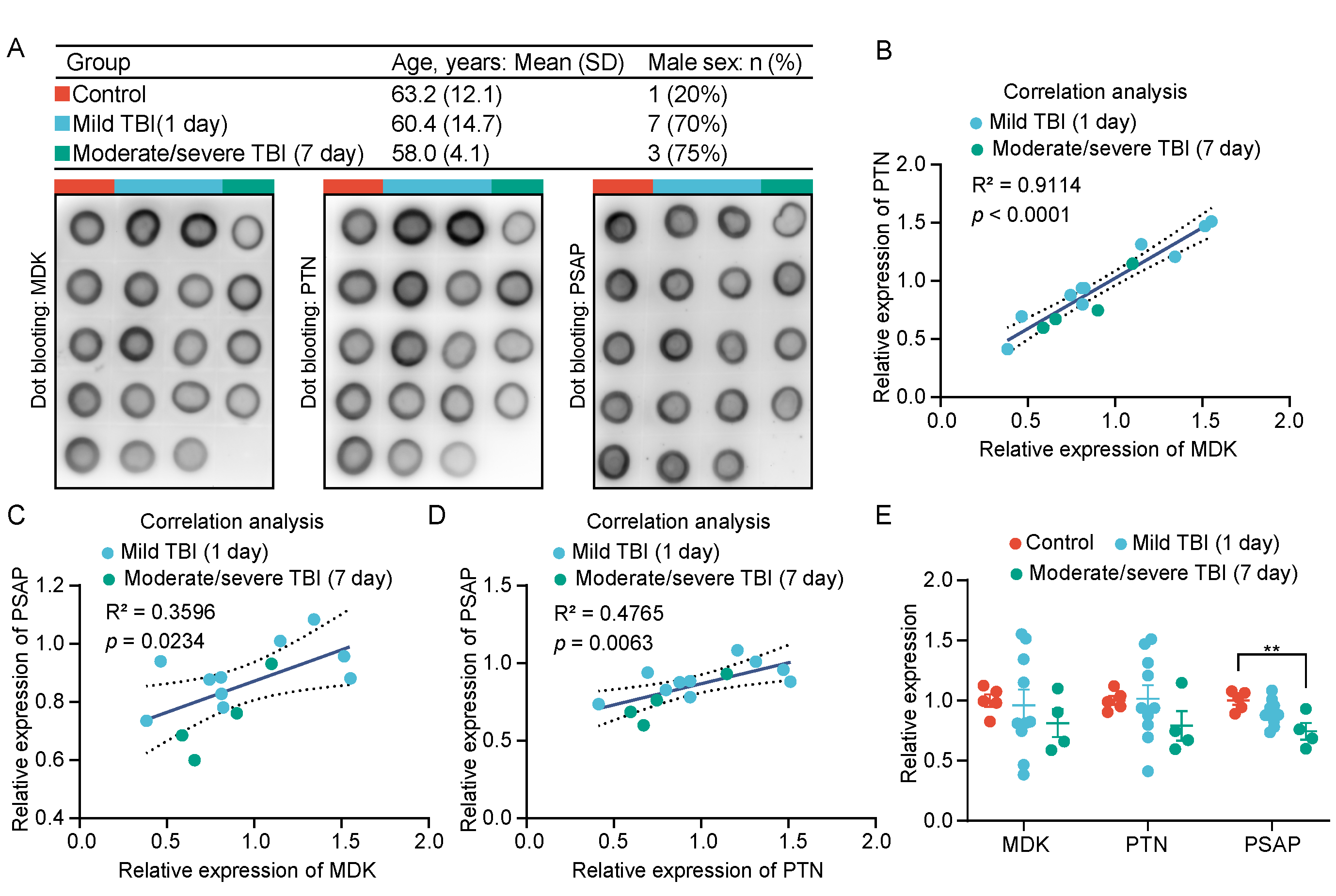

Supplement: Supplementary file 8 — Figure S4. [file CNS-29-3446-s004.tiff]

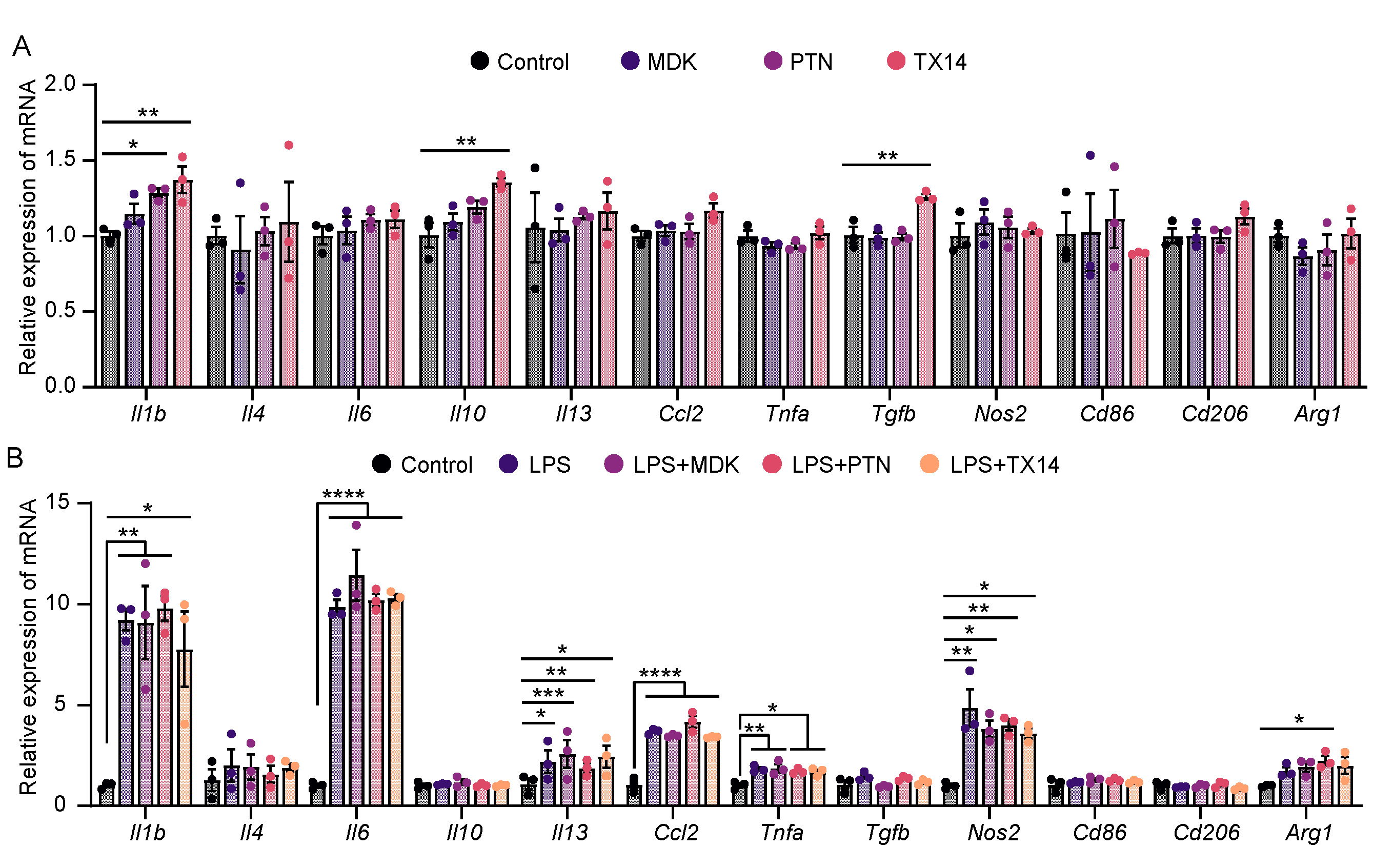

Supplement: Supplementary file 9 — Figure S5. [file CNS-29-3446-s003.tiff]
